# Adapting camera‐trap placement based on animal behavior for rapid detection: A focus on the Endangered, white‐bellied pangolin (*Phataginus tricuspis*)

**DOI:** 10.1002/ece3.10064

**Published:** 2023-05-10

**Authors:** Franklin T. Simo, Ghislain F. Difouo, Sévilor Kekeunou, Ichu G. Ichu, David Olson, Nicolas J. Deere, Daniel J. Ingram

**Affiliations:** ^1^ Laboratory of Zoology, Department of Biology and Animal Physiology University of Yaoundé I Yaoundé Cameroon; ^2^ Cameroon Wildlife Conservation Initiative (CWCI) Yaoundé Cameroon; ^3^ IUCN SSC Pangolin Specialist Group, ℅ Zoological Society of London London UK; ^4^ Carnivore and Population Ecology Laboratory, Department of Wildlife Fisheries and Aquaculture Mississippi State University Mississippi State Mississippi USA; ^5^ NEOM Nature Reserve Gayal Kingdom of Saudi Arabia; ^6^ Durrell Institute of Conservation and Ecology, School of Anthropology and Conservation University of Kent Canterbury UK

**Keywords:** Africa, Deng Deng National Park, monitoring, Mpem et Djim National Park, *Pholidota*

## Abstract

Pangolin species are notoriously difficult to detect and monitor in the wild and, as a result, commonly used survey techniques fall short in gathering sufficient data to draw confident conclusions on pangolin populations, conservation status, and natural history. The white‐bellied pangolin is a semiarboreal species that may be poorly detected in general mammal surveys even with modern techniques such as camera‐trapping. As a result, population status information is often derived from hunting, market, and trafficking data. There is therefore a crucial need to improve camera‐trap survey methods to reliably detect this species in its natural environment. Here, we test the influence of camera‐trap placement strategy on the detectability of the white‐bellied pangolin by comparing estimates from targeted ground‐viewing camera‐trapping and a novel log‐viewing placement strategy adapted from local hunters' knowledge. Our results suggest that (1) deploying camera‐traps to detect animals walking along logs is an effective strategy for recording several forest species, including the white‐bellied pangolin, and (2) that camera‐traps targeting logs are more efficient at detecting white‐bellied pangolins than camera‐traps viewing the ground (>100% increase in detection probability). We also found moderate evidence that there is a relationship between the white‐bellied pangolin occurrence at our locality and elevation and weak evidence of an association with distance to the nearest river. Our results suggest an effective new monitoring approach allowing consistent detection of the white‐bellied pangolin with moderate survey effort. This highlights the importance of harnessing local knowledge to guide the design of monitoring protocols for cryptic species.

## Graphical abstract

Pangolin species are notoriously difficult to detect and monitor in the environment. As a result, regularly used survey methodologies fail to acquire enough data to make reliable conclusions on their numbers, conservation status, and natural history. Here, we test the influence of camera‐trap placement strategy on the detectability of the white‐bellied pangolin by comparing estimates from targeted ground‐viewing camera‐trapping and a novel log‐viewing placement strategy adapted from local hunters' knowledge. Our results suggest that (1) deploying camera‐traps to detect animals walking along logs is an effective strategy for recording several forest species, including the white‐bellied pangolin, and (2) that camera‐traps targeting logs are more efficient at detecting white‐bellied pangolins than camera‐traps viewing the ground (>100% increase in detection probability).

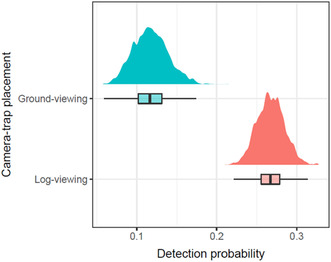

## 1 INTRODUCTION

Pangolins form a group of mammals whose morphology and lifestyle make it challenging to study and monitor in the wild using conventional survey methods (Ingram et al., 2019; Willcox et al., 2019). They are largely nocturnal, solitary, and most species occur at naturally low densities (Jansen et al., 2020; Kingdon, 1988). Two African pangolin species, the giant pangolin *Smutsia gigantea* (Illiger, 1815) and Temminck's pangolin *Smutsia temminckii*, (Smuts, 1832) are ground‐dwelling and use subterranean burrows. The smaller white‐bellied *Phataginus tricuspis* (Rafinesque, 1821) and black‐bellied pangolin *Phataginus tetradactyla* (Linnaeus, 1766) are semiarboreal (scansorial) and arboreal, respectively, which makes them harder to detect using terrestrial camera‐trap surveys.

The white‐bellied pangolin can be found in trees, in tree cavities, and on the ground (Akpona et al., 2008; Jansen et al., 2020). Although this species is regularly recorded in mammal inventories employing 'ground‐viewing' camera‐traps, such inventories typically produce only a few observations of pangolins, too few, at times, to confidently analyze detection and population trends, and how they are shaped by environmental and anthropogenic variables (Bruce et al., 2018; Lahkar et al., 2018; Moo et al., 2018).

The likelihood of recording a species at a given location is a function of the species' abundance and movement pattern (Kolowski & Forrester, 2017; Sollmann et al., 2013), but a wide range of other parameters (e.g., habitat, camera setup, researcher experience) can also have significant effects on the detectability of a species (Sollmann et al., 2013). Cusack et al. (2015) highlighted the considerable influence that camera‐trap placement can have on a species' detection probability. While there is evidence that some mammal species, such as carnivores, may display a preference for using trails and human tracks (Kays et al., 2010; Kelly & Holub, 2008; Sollmann et al., 2013; Wearn et al., 2013), there is no published evidence that the ground‐utilising species of pangolins have any preferences for traveling routes in their habitats that could inform where to deploy camera‐traps to maximize detections.

The white‐bellied pangolin is categorized as Endangered on The IUCN Red List of Threatened Species (Jansen et al., 2020) and classified as Class A species under the national legislation in Cameroon (TRAFFIC, 2019). The Order Pholidota has been shown to have the highest percentage of species threatened by hunting of all mammalian Orders (Ripple et al., 2016). Local hunters in the Cameroonian forest‐savanna mosaic zone state that white‐bellied pangolin are frequently observed on trees (alive and fallen), often using fallen logs to traverse across the forest floor (Difouo et al., 2020). A popular hunting strategy among these hunters includes setting homemade traps on top of fallen trees to increase their chances of catching white‐bellied pangolin (Simo et al., 2023). Camera‐traps targeting fallen logs have been shown to produce significant improvements in the detection of several North American focal species, including arboreal species (Kolowski & Forrester, 2017). A preliminary camera‐trapping survey targeting several types of possible pangolin activity sites identified by local guides' shows that targeting logs initially appears to be a useful tactic to increase chances of detecting the presence of white‐bellied pangolins in tropical forest habitats (Simo et al., 2020). Ichu (2022) also used a log‐based placement to record the white‐bellied pangolin in Cameroon but did not specifically look at the difference with traditional ground‐viewing camera‐trapping. Here, we adopt an occupancy modeling framework to explicitly compare detection probability of white‐bellied pangolins using two different targeted camera‐trap placement strategies, where camera‐traps are (1) placed to detect animals at ground level (‘ground‐viewing’) and (2) placed to detect animals walking along the tops of fallen logs (hereafter ‘log‐viewing’). We investigate how detection of the white‐bellied pangolin is shaped by environmental covariates. We also compare the overall species richness and detections between the two camera‐trapping methods for the entire mammal community to determine whether the alternative 'log‐viewing' placement, if implemented, is likely to result in differences in the detections of other taxa.

## 2 METHODS

### 2.1 Study area

**FIGURE 1:**
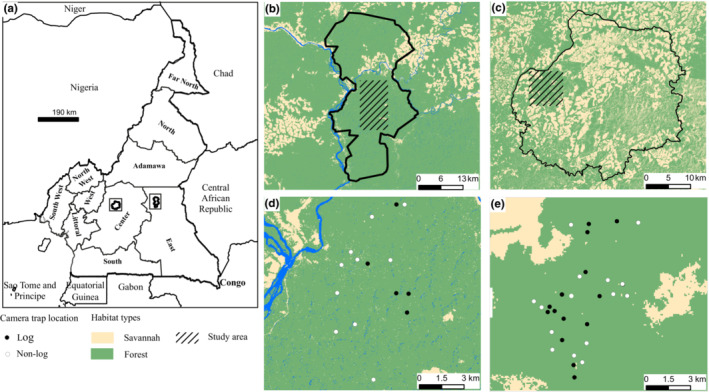
Map of the study area showing the deployment stations within the forested area of each national park. (a) Location of Deng Deng and Mpem et Djim National Parks (NPs) in Cameroon; (b, c) map of Deng Deng and Mpem et Djim NPs showing the survey area; camera‐trap stations in the forested area of Deng Deng and Mpem et Djim NPs, showing ground‐viewing stations (white circles) and log‐viewing stations (black circles). Habitat type was mapped using a supervised classification approach in ERDAS IMAGINE 2014 and ArcGIS 10.6.

This study was carried out in Deng Deng National Park (5°‐5°25′ N, 13°‐23°34′ E; 682 km^2^; average altitude: 703 m; Figure 1a,b) and in Mpem et Djim National Park (5°‐5°20′ N/11°30′‐12°E; 976 km^2^; average altitude of 640 m; Figure 1a,c) situated in the Eastern (Lom et Djerem Division) and Centre Region (Ntui Division) of Cameroon, respectively. Deng Deng and Mpem et Djim National Parks (NPs) are located in the transitional zone of Cameroon and have a mix of closed‐canopy forests, savannah grasslands, and gallery forests, sustaining both forest and savannah‐dwelling species. The climate is a classic Guinean type with four seasons: a long dry season (from mid‐November to mid‐March); a short rainy season mid‐March to end of June; a short dry season (from July to August) and a long rainy season (from September to mid‐November; Tsalefac et al., 2003).

### 2.2 Camera‐trapping

**FIGURE 2:**
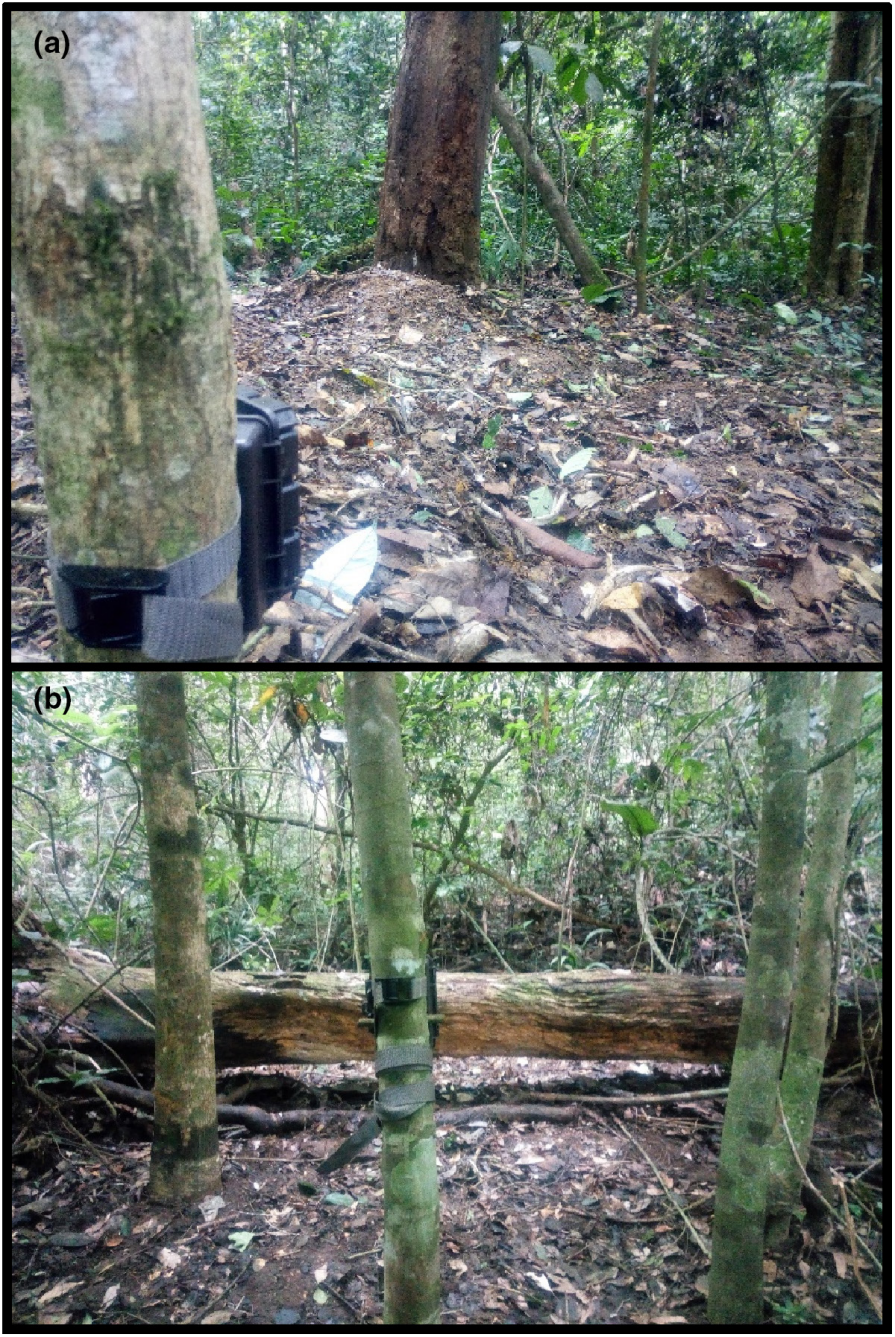
Targeted camera‐trap placement deployed at suspected activity sites of the white‐bellied pangolin using (a) ground‐viewing placement and (b) log‐viewing placement.

We deployed 45 camera‐traps in the forested area (closed‐canopy forest) of Deng Deng and Mpem et Djim NPs. Twenty‐five camera‐traps were deployed to view the ground (Figure 2a) and 20 were deployed to target the upper surfaces of logs (i.e., dead fallen tree trunks; Figure 2b). A camera‐trap was deployed at each station during both the rainy and dry seasons (Table 1), and each station was monitored for 3 months to culminate in a minimum survey effort of 1000 camera‐trap days for each survey as suggested by Tobler et al. (2008). The camera‐traps were placed perpendicular to the targets at a regular distance of 4 m to get full‐body lateral images of the animal (Ahumada et al., 2011; Amin et al., 2015). Camera‐traps were set to the maximum sensitivity and programmed to shoot three photographs with the minimum possible time elapsed between subsequent triggers. We took precautions to reduce the chance of factors other than the placement strategy influencing species detection. For example, all camera‐traps were of the same model (Bushnell Trophy Camera Brown 119,836) equipped with the same SD cards (SanDisk 16 GB class 10, 48 MB/s). All cameras were deployed in the same habitat type (closed‐canopy forest) to avoid habitat bias. Camera‐trapping was conducted by the same group of individuals and cameras were installed by the lead author alone, to reduce bias arising from researcher's experience.

**TABLE 1:** Survey period and camera‐trapping effort according to protected area and season.

Season	Deng Deng NP		Mpem et Djim NP	
	Rainy	Dry	Rainy	Dry
Survey from	April 2018	December 2019	May 2019	December 2019
Survey to	June 2018	April 2020	September 2019	March 2020
Sampling effort	886	1056	2626	2494
N° camera stations for Ground placements	10	10	15	15
N° camera stations for Log placements	5	5	15	15

'Ground‐viewing' cameras were strapped to trees at a height of 30–40 cm above the ground level, suitable for small‐to‐medium‐sized mammals (Amin et al., 2015; Bruce et al., 2018). These camera‐traps were placed at ground‐level areas showing visible/suspected signs of the white‐bellied pangolin activities, namely feeding sites on trees, feeding sites on the ground, burrows in the ground, and cavities in trees (see Simo et al., 2020 for details of these sites).

'Log‐viewing' camera‐traps were placed so that they were pointed at fallen logs and were deployed at different heights according to the adjacent tree trunk diameter, but all camera‐traps were 30 cm above the top of the fallen log (Simo et al., 2020). Selection of fallen tree trunks at which camera‐traps were pointed was based on the presence of signs of animal activities on the upper side, such as scat, scratches, disturbed surfaces, and excavations. No size criteria were applied for selection of tree trunks; however, the trunks selected were deemed large enough to allow animal passage. We utilized a long‐range battery palmtop for the in situ checking of all camera‐trap placement, this included verifying if the trunk was in the camera's field of view and adjusting angling and positioning.

### 2.3 Analyses

We considered independent events as sequential images of clearly distinct individuals taken at the same location (e.g., different species, different body sizes). In cases where the same species triggered the same camera at an interval of <1 h, we considered these consecutive records as a single detection and used only the time of the initial trigger (Bowkett et al., 2009; Rowcliffe et al., 2014; Tobler et al., 2008). The encounter rate (ER) was calculated by dividing the number of notionally independent events by the number of trap days [the number of 24‐hour periods in which cameras were in operation until they were retrieved (Bruce et al., 2018; Jenks et al., 2011)] and multiplied by 100, to give estimates per sampling effort of 100 days.

Prior to occupancy analysis, we prepared our dataset to satisfy certain modeling assumptions. To ensure independence between trapping stations, five camera‐trap stations (three ‘log‐viewing’ and two ‘ground‐viewing‘ cameras) were excluded from our dataset to ensure that those remaining were at least 500 m apart to reduce the possibility of autocorrelation between camera stations. This distance was chosen based on the estimated species home range (Pagès, 1975). We then constructed a detection/nondetection history for each camera‐trap station, denoting the presence or absence of the focal taxa across sampling occasions comprising five camera‐trap days. Initial assessment indicated that occupancy and detection were statistically comparable between rainy and dry seasons; therefore, detection data were aggregated into a single season for the analysis.

To investigate possible links between the white‐bellied pangolin detection and occupancy and the different placement strategies and environmental predictor variables, respectively, we implemented a Bayesian single‐species, single‐season occupancy model. Occupancy models capitalize on temporally replicated samples to separate the state process, describing the partially observed ecological quantity of interest (occurrence), from the observation process (detection) underpinning the data (Guillera‐Arroita, 2017; MacKenzie et al., 2002). This hierarchical specification accounts for bias associated with imperfect detection and allows explicit consideration of factors influencing the probability of detecting a species given that it occupies a sampling location. To test the effect of camera‐trap placement on the detection of white‐bellied pangolins, we included placement as a fixed intercept in the detection part of the model. We also included a survey effort covariate on the detection component of the model to account for differences in occasion length due to camera‐trap malfunction. The occupancy component tested the effects of the following environmental variables on pangolin occupancy: distance to river or stream, distance to protected area (PA) boundary, and elevation, which were automatically generated using GIS data of the park in ArcMap and season (dry or rainy), generated from the survey period. Preliminary analyses indicated that there was no substantial difference in baseline pangolin occupancy estimates between the two national parks, so PA‐specific intercepts were not included within the modeling framework.

To demonstrate the potential benefits of directed camera‐trap placement for survey design, we practically applied model‐derived occupancy (*ψ*) and detection (*p*) probabilities to estimate the required number of camera stations (*s*) to achieve a standard error (var(*ψ*)) within 10% of the mean occupancy estimate (i.e., var(*ψ*) = 0.05), using the formula (Mackenzie & Royle, 2005)

$$s=\frac{\psi }{\mathrm{var}\left(\hat{\psi }\right)}\left[\left(1-\psi \right)+\frac{\left(1-p^{*}\right)}{\;p^{*}-\mathrm{Kp}{\left(1-p\right)}^{K-1}}\right]$$

where p* denotes the probability of detecting the focal species during at least one of *K* sampling occasions. Throughout, we fix *K* to a value of 12, corresponding to a 60‐day deployment based on optimum recommendations for tropical applications of camera‐trap methods (Kays et al., 2020).

Bayesian occupancy analysis was conducted in R 4.2.1 (R Core Team, 2020), but implemented using JAGS 4.3.1 (Plummer, 2003) through *jagsUI* 1.5.2 (Kellner & Meredith, 2021). To ensure adequate characterization of the posterior distribution of our estimates, we specified three Markov chain Monte Carlo (MCMC) chains, each comprising 50,000 iterations and a burn‐in and thinning rate of 10,000 and 40, respectively. We first assessed model convergence through visual inspection of trace plots. Then, we checked the Gelman–Rubin convergence diagnostic (R‐hat; Gelman & Hill, 2006) in our models, which indicated adequate mixing for all estimated parameters (R‐hat <1.1). Statistical associations with predictor variables were considered to be substantial and moderate when the 95% Bayesian credible interval (95% BCI; 2.5th and 97.5th percentiles of the posterior distribution) and 75% BCI (12.5th and 87.5th percentiles) of their effect sizes, respectively, did not overlap zero.

## 3 RESULTS

### 3.1 Species recorded using ‘ground‐viewing’ vs ‘log‐viewing’ camera‐trapping

**FIGURE 3:**
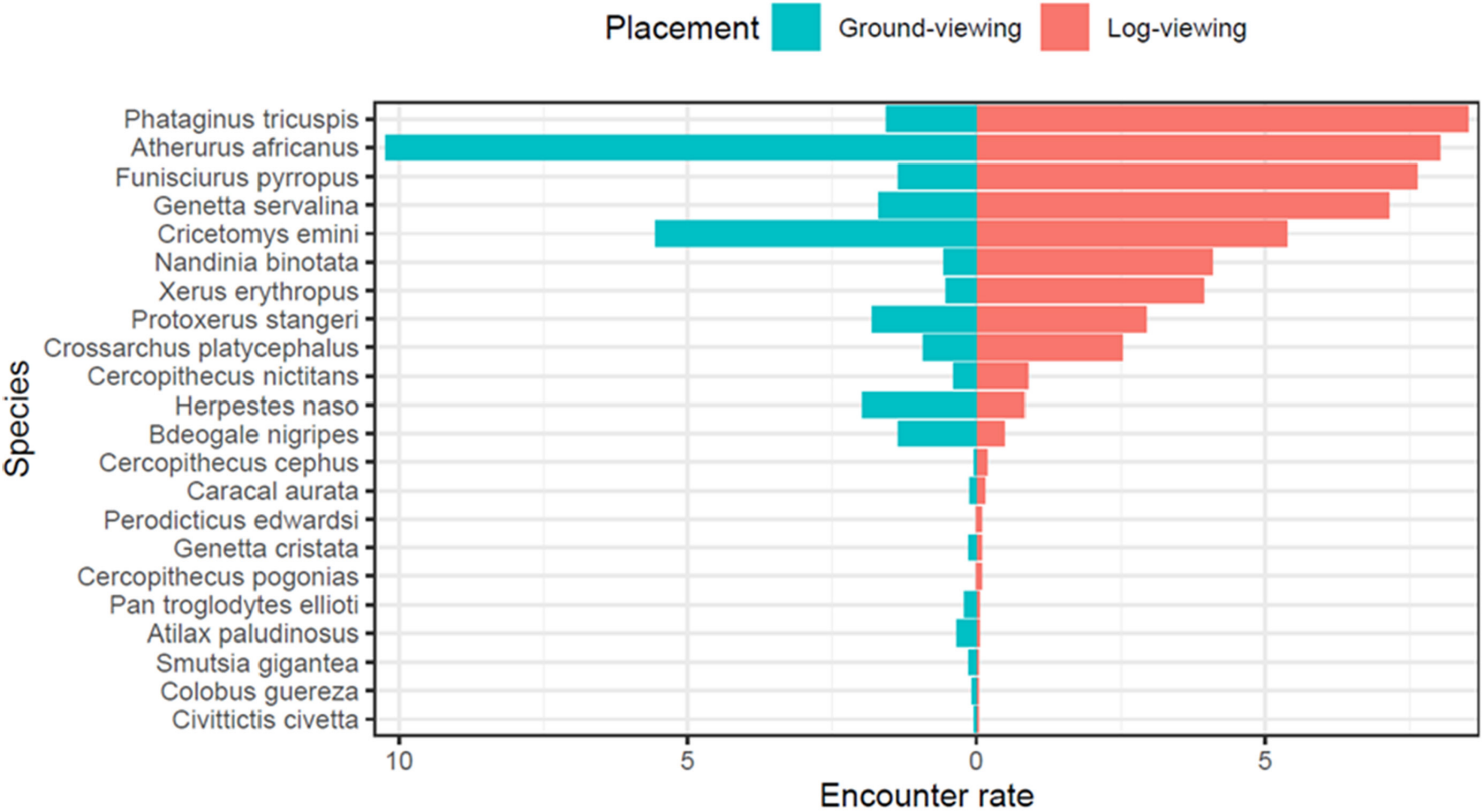
Encounter rate (ER) of species recorded on camera‐traps placed using a log‐viewing strategy, plotted against their encounter rate from the targeted ground‐viewing camera‐trapping strategy. Comparisons are from species that were recorded on top of logs during the log‐viewing camera‐trap surveys only. ER = the number of independent events of a species divided by the number of trap days (per placement strategy) and multiplied by 100.

We recorded a total of 33 mammal species (28 in Deng Deng NP and 26 in Mpem et Djim NP). A total of 30 species were recorded on ‘ground‐viewing’ camera‐trap stations compared with 32 species recorded at ‘log‐viewing’ camera‐traps. Of the species recorded at ‘log‐viewing’ stations, 22 (68.75%) were recorded traveling on top of the logs (Figure 3), while 10 (31.25%) were recorded either not on logs or detected when crossing over the logs. The latter includes ungulate species and great apes. Bushbuck, *Tragelaphus scriptus* were photographed at ground‐viewing stations but were never recorded at log‐viewing stations. White‐bellied pangolin had the highest capture rate among the species recorded traveling on logs. Milne‐Edwards's Potto *Perodicticus edwardsi* and Crested mona monkey *Cercopithecus pogonias* were photographed on logs but were never recorded on the ground‐viewing camera‐traps.

### 3.2 Comparison of detection between ‘ground‐viewing’ and ‘log‐viewing’ placement

Overall, we detected a total of 338 pangolin events from 7062 camera‐trap days accumulated across both parks, seasons, and placement types (Table 2). We obtained a trapping rate of 4.78 per 100 trap days for the whole area. The white‐bellied pangolin was recorded at 57 of the 88 locations monitored, which includes 36 of the 39 ‘log‐viewing’ stations monitored (92.3%) and 21 of the 49 ‘ground‐viewing’ stations monitored (42.9%). A survey effort of 3280 camera‐trap days recorded 279 white‐bellied pangolin events on ‘log‐viewing’ camera‐traps (ER = 8.5) while a survey effort of 3782 camera‐trap days recorded 59 white‐bellied pangolin events on ‘ground‐viewing’ camera‐traps (ER = 1.56). The shortest delay for recording a pangolin on a ‘log‐viewing’ camera‐trap was <1 day, where a camera in Deng Deng NP recorded a pangolin the day it was installed (average number of days = 34.12). The shortest delay for recording a pangolin on a ‘ground‐viewing’ camera‐trap was 2 days (average number of days = 48.67).

**TABLE 2:** Summary of the number of surveyed stations, the sampling effort, and the detection rate per placement strategy.

White‐bellied pangolin	Log	Ground	Total
Number of survey stations	39	49	88
Number of stations detected	36	21	57
Survey effort	3280	3782	7062
Number of events	279	59	338
Number of photos	1180	404	1584
Encounter rate per 100 days	8.5	1.56	4.78

### 3.3 Detection and occupancy analysis

**FIGURE 4:**
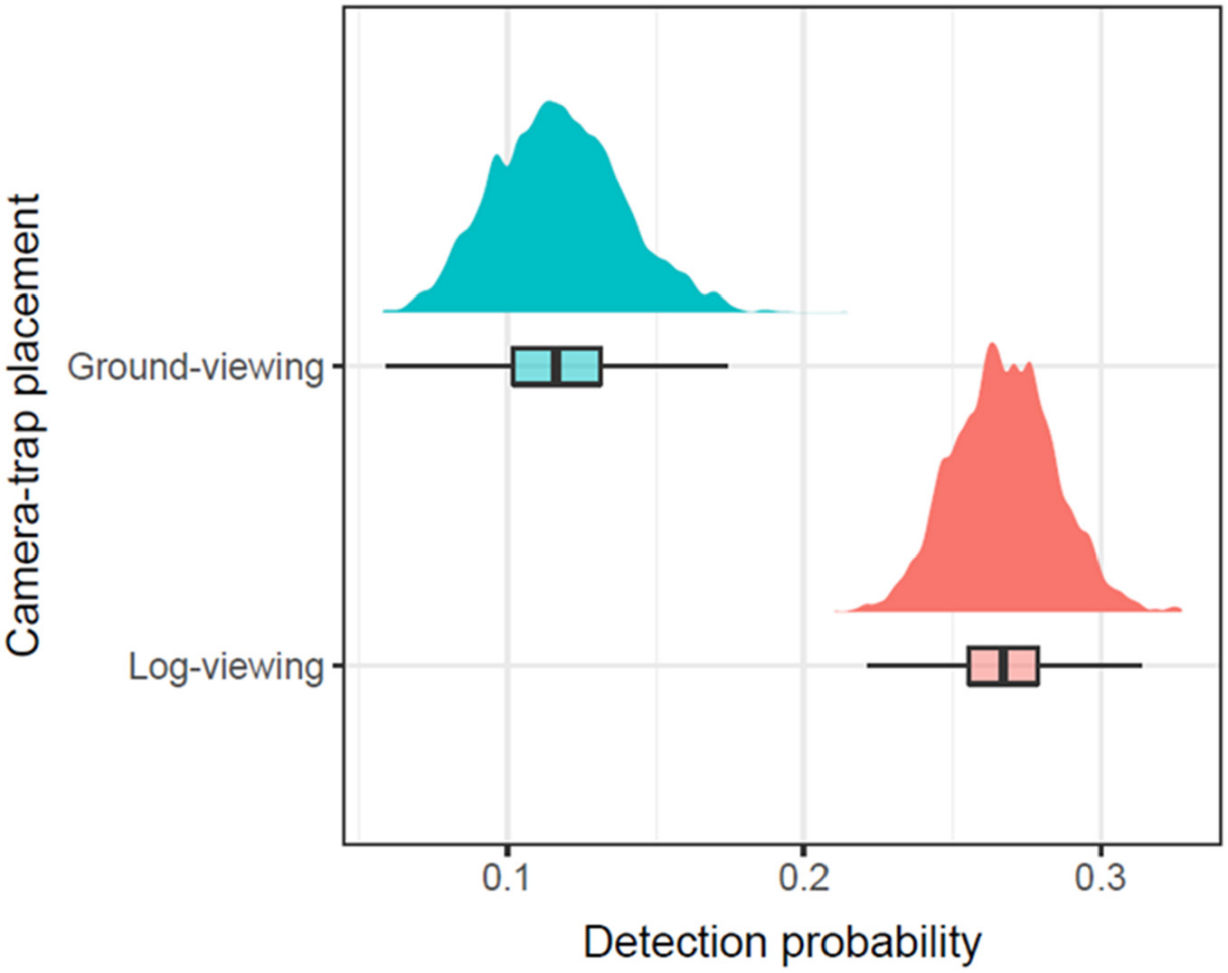
Bayesian occupancy model results showing the effect of camera‐trap placement on the detection probability of white‐bellied pangolins in two national parks in Cameroon. ‘Ground‐viewing’ refers to camera‐traps placed at ground level, while ‘log‐viewing’ refers to camera‐traps placed to view the tops of fallen trees.

**FIGURE 5:**
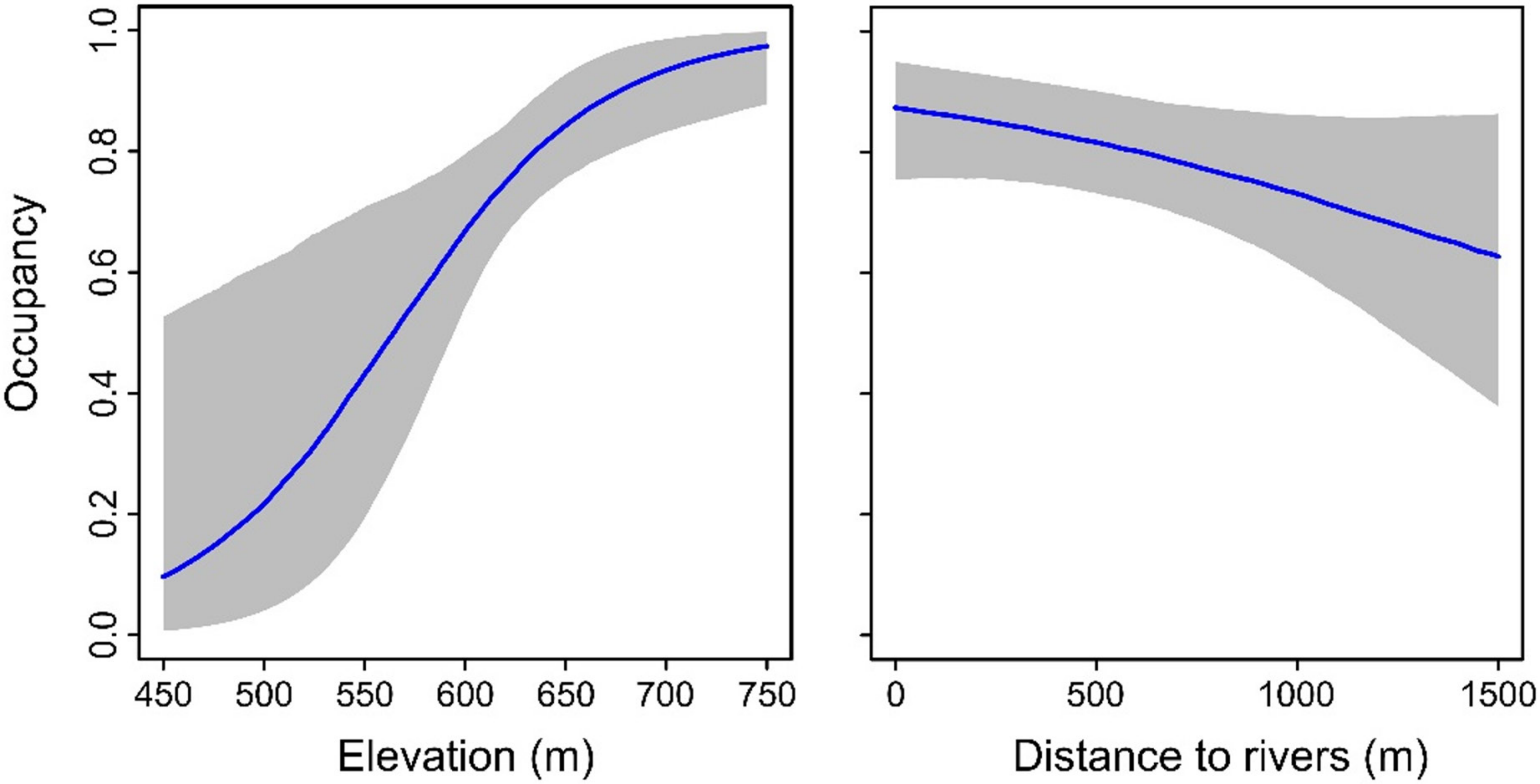
Predicted occupancy of the white‐bellied pangolin in relation to elevation (left) and distance to the nearest river (right). The evidence for a relationship between pangolin occupancy and elevation was moderate (75% Bayesian Credible Interval; BCI) and with distance to rivers was very weak (50% BCI). The shaded polygon represents the 75% confidence interval surrounding the regression line.

Quadratic terms of predictor variables were not found to be influential, so were excluded in the final model. There was strong evidence from our model that log‐viewing placement is more effective at detecting white‐bellied pangolin posterior mean = 0.26; 95% BCI = 0.23–0.30 than ground‐viewing placement (0.12; 0.08–0.16; Figure 4). We also found moderate evidence that elevation has a positive influence on pangolin occupancy (at the 75% BCI level) and weak evidence that distance to rivers has a negative association at the 50% level (Figure 5). We found no effect of distance to PA boundary on pangolin occupancy.

Based on a model‐derived occupancy probability of 0.80 (95% BCI: 0.66–0.96), 224 camera stations (130–537) would be required to achieve precise occupancy estimates within 10% of the true population value when units are deployed using traditional ground‐based placement. Directed camera placement toward fallen logs could potentially reduce survey effort by 67% on average, with only 74 camera stations (69–91) required to estimate pangolin occurrence with a high level of precision.

## 4 DISCUSSION

Local hunters in our study area stated that white‐bellied pangolins commonly used fallen trees to traverse the forest which was later confirmed when white‐bellied pangolins were regularly documented on top of logs during a prior camera‐trapping survey in Deng Deng NP (Simo et al., 2020). For these reasons, we tested and compared the effectiveness of a targeted ground‐viewing camera‐trap placement against a log‐viewing placement strategy to detect white‐bellied pangolins. Placement of camera‐traps viewing logs significantly increased the detection probability of white‐bellied pangolins, being more than double that of the ground‐viewing camera‐trap placement.

Our study demonstrates the utility of targeting small‐scale habitat features, such as logs on the detectability of the white‐bellied pangolin as compared to ‘ground‐viewing’ camera‐trapping, in a design that controlled or minimized the impact of external factors on detection probability. Using our adapted camera‐trap placement, 100 detections could be achieved in less than 1200 camera‐trap days, which corresponds to using 20 camera‐traps each deployed on logs for 60 days. This ‘log‐viewing’ camera‐trap approach has been shown to be more effective at recording the white‐bellied pangolin in a study that attempted to compare different detection methods including a ‘log‐viewing’ camera placement and an arboreal placement strategy in Campo Ma'an NP in southern Cameroon (Ichu, 2022). Thus, ‘log‐viewing’ camera‐trap surveys may be a cost‐effective and recommendable method for detecting the white‐bellied pangolin.

White‐bellied pangolin occupancy was positively associated with elevation between 450 and 750 m, and possibly negatively associated with increasing distance to rivers (although there was only weak evidence for the latter), which is in line with the current understanding of the white‐bellied pangolin ecology (Jansen et al., 2020; Kingdon et al., 2013). Khwaja and colleagues (2019) observed a decreasing probability of occupancy with increasing elevation, which contrasts with the trend observed in our results. While we found no effect of distance to PA boundary on pangolin occupancy in our study, Bruce et al. (2018) observed a decreasing and an increasing trend in occupancy probability with distance to park boundary in two different management sectors of the Dja Faunal Reserve, possibly showing contrasting levels of hunting pressure. Log diameter does not appear to influence the detection probability for any species at 'log‐viewing' cameras (Kolowski & Forrester, 2017). However, this study was for eastern North America only and the impact of log diameter on detection probability outside of that region is unknown. Furthermore, evaluation of other potential factors that could influence detection, such as the decaying state of the logs, proximity to feeding sites, and the amount of vegetation covering the logs, is warranted.

Our study also recorded several other species using logs as travel routes, such as the African golden cat *Caracal aurata* which had five out of nine African golden cat detections on logs. This may be because traversing logs may be a good hunting strategy either because it facilitates travel in dense understory or because it reduces noise and the likelihood of being detected by prey species (see Simo et al., 2020). Logs have also been shown to act as ecological corridors for wildlife, connecting terrestrial habitats across streams (Trevarrow & Arismendi, 2022). As compared to the ground‐viewing strategy, our log‐viewing placement strategy also yielded more detections for many other species. These include the African palm civet *Nandinia binotata*, the African ground squirrel *Xerus erythropus*, the Cameroon cusimanse *Crossarchus platycephalus*, the forest giant squirrel *Protoxerus stangeri*, the red‐legged rope squirrel *Funisciurus pyrropus*, and the servaline genet *Genetta servalina*. Furthermore, by placing cameras perpendicular to the logs, we were able to get high‐quality lateral images of animals walking on logs, which may help with species and individual animal identification. Although employing the log‐viewing placement strategy resulted in fewer detections for great apes and ungulates in our study, it recorded more species than the alternative ground‐viewing camera‐trap placement, as also observed by Kolowski and Forrester (2017). For future camera‐trap studies for which presence, richness, and diversity are sought measures, we recommend that including multiple microhabitats (including log “highways”) may avoid underestimates of species' presence or pseudo‐absences (Cusack et al., 2015; Kolowski & Forrester, 2017).

Our study employed an opportunistic camera‐trap design rather than the systematic design often used for occupancy modeling. This was due to the uncertainty of finding a suitable location that met our selection criteria for camera‐trap deployment. Given that the home range of the white‐bellied pangolin is estimated to be in the range of 500 m^2^ (Kingdon et al., 2013; Pagès, 1975), we excluded in our analysis camera‐traps that were less than 500 m apart to satisfy the modeling assumption of independent observations, although the home range may vary considerably between parameters, such as pangolin sex, seasons, sites, habitat types, and level of hunter offtake. While we did not employ a paired camera design, the common practice for comparing detection from camera‐traps between different placements (Cusack et al., 2015; Kolowski & Forrester, 2017), deploying our cameras in the same habitat type was important to limit biases that can arise from habitat heterogeneity.

Our study employed both occupancy modeling and the ER as metrics to compare between placement types. While the ER metric is limited for comparisons between different species due to a wide range of biases, comparing ERs of a single species throughout time and/or space is suitable because factors confounding the imperfect detection between different species are expected to decrease within the same species (Sollmann et al., 2013). ER in our results is interpreted as an index of activity, where activity of the white‐bellied pangolin is thought to be higher on logs in comparison with on the ground. In this context, applying an occupancy model is helpful to understand relationships between our predictor variables and white‐bellied pangolin occupancy, while testing different camera placement strategies (see Sollmann, 2018).

Although the white‐bellied pangolin is the most frequently encountered of all the African pangolin species and is seemingly capable of occurring in moderate densities in appropriate habitats and sites where hunting occurs (Kingdon et al., 2013), its semiarboreal nature combined with its relatively small size makes it difficult to document on camera‐traps, regardless of their local densities, and recent studies even indicate a declined availability of this species (Difouo et al., 2020; Mouafo et al., 2021; USAID/WA BiCC, 2020). Hence, an improved strategy for recording the white‐bellied pangolin, like the one we present here, is crucial for accurate assessments of its conservation status. While no camera‐trap studies have yet investigated pangolin density, the enhanced detection that our method provides could potentially be integrated into methods that account for non‐individually recognizable species (Howe et al., 2017; Moeller et al., 2018), to produce meaningful density estimates of pangolin. Finally, as demonstrated previously in Simo et al. (2020), the method and results presented here highlight the importance of harnessing local knowledge to guide monitoring approaches of cryptic species but also open new research possibilities for population monitoring of the white‐bellied pangolin.

## AUTHOR CONTRIBUTIONS

**Franklin T. Simo:** Conceptualization (lead); data curation (lead); formal analysis (lead); funding acquisition (lead); investigation (lead); methodology (lead); project administration (lead); supervision (equal); validation (equal); visualization (equal); writing – original draft (lead); writing – review and editing (lead). **Ghislain F. Difouo:** Conceptualization (equal); data curation (equal); funding acquisition (equal); investigation (equal); methodology (equal); project administration (equal). **Sevilor Kekeunou:** Conceptualization (equal); data curation (equal); supervision (lead); validation (equal). **Ichu G. Ichu:** Conceptualization (equal); data curation (equal); funding acquisition (equal). **David Olson:** Conceptualization (lead); supervision (lead); validation (lead); writing – review and editing (equal). **Nicolas Deere:** Formal analysis (equal); validation (equal); writing – review and editing (supporting). **Daniel J. Ingram:** Conceptualization (equal); Formal analysis (equal); supervision (lead); validation (equal); visualization (equal); writing – review and editing (equal).

## FUNDING INFORMATION

This research received financial support from the Rufford Small Grant Foundation (Award number): and Conservation Action Research Network (CARN). DJI acknowledges support from UK Research and Innovation (Future Leaders Fellowship awarded to DJI, Grant ref: MR/W006316/1)

## CONFLICT OF INTEREST STATEMENT

The authors declare no conflicts of interest.

## Data Availability

All data and code used to conduct the analysis reported in this paper are available through the Dryad Digital Repository: DOI https://doi.org/10.5061/dryard.pk0p2ngt2. (Simo et al., 2023)
